# Supplementary material for: Yeasts Have Evolved Divergent Enzyme Strategies To Deconstruct and Metabolize Xylan
Source: Microbiol Spectr. 2023 Apr 26;11(3):e00245-23. doi: 10.1128/spectrum.00245-23 (PMC10269524; doi:10.1128/spectrum.00245-23)
Supplement: Supplemental file 3 — List S1, Tables S1 and S2, and Fig. S1 to S8. Download spectrum.00245-23-s0001.docx, DOCX file, 4.7 MB [file spectrum.00245-23-s0001.docx]

# Supplemental information

**Yeasts have evolved divergent enzyme strategies to deconstruct and metabolize xylan**

Jonas L. Ravn, Amanda Sörensen Ristinmaa, Tom Coleman, Johan Larsbrink, and Cecilia Geijer

**List S1. Gene sequences extracted from automated genome prediction.** Zenodo link: <https://zenodo.org/record/4548336#.YvTiwHZByUk>

***Bm*Xyn11A** (*Blastobotrys mokoenaii*)

Gene ID: ID_129-processed-gene-0.19

>snap_masked-NODE_65_length_31672_cov_37.998_ID_129-processed-gene-0.19-mRNA-1 gene=snap_masked-NODE_65_length_31672_cov_37.998_ID_129-processed-gene-0.19 CDS=1-651 **ATGAAACTTTCCAACGCCATAACTGCAATTTGCGCTGCCGCTGTCCTCGCT**GCTCCATTGGAGGAGGAAGAGGTAGCTAAGCGAAGTGTAACTCCATCTAGCACTGGTACTAACAACGGCTACTATTATTCCTTCTGGTCTGACGGTGGTGGAGATGTTACCTACACCAACGGAAATGGTGGCTCCTACAGTGTGGAATGGACCAACTGCGGTAACTTTGTAGGTGGAAAGGGCTGGAATCCAGGTGCTGCTCGGGAAATCAACTTTTCTGGCTCCTTCAATCCTTCAGGCAATGGCTACCTCTCTGTCTACGGCTGGACTACCAACCCACTTGTAGAGTACTACATTGTTGAGTCCTATGGCGACTACAACCCCGGAACTGCGGGAACCTTCCTCGGTACTGTTGATTCCGATGGTTCGACATATGATATCTATAAGGCTGTTCGAACTAATGCGCCATCTATTGAGGGTACGGCTACATTTGACCAGTACTGGTCTATCAGACGAAATCACCGAACTAGCGGTACTGTTAACACCGGCAACCATTTCAACGCCTGGGCTCAGCATGGTCTGCAACTCGGTACTCACAATTACCAGATTGTTGCCACTGAGGGATATCAGAGCTCTGGTTCGTCCTCTATCACTGTTTCT

(Bold bases: predicted signal peptide)

***Bm*GH30_7** (*Blastobotrys mokoenaii*)

Gene ID: ID_61-processed-gene-0.204

>genemark-NODE_31_length_153071_cov_36.322_ID_61-processed-gene-0.204-mRNA-1 gene=genemark-NODE_31_length_153071_cov_36.322_ID_61-processed-gene-0.204 CDS=1-1152 ATGAATTCAATTGAACCATTTAGCCCTGGGTCCCCTAGCAATCCACCTCATTACACATGGGACAATTATGACAGTGGACAATTACAGCTTGCAAAAGAAGCCAAGAAGCGAGGCTTAGCGACCCTTTATGCTGATGCATGGTCGGCTCCAGGATATATGAAGTCCAACAATGATGAGAATTGGGGCGGTTATCTTTGCGGTGTCTCAGGAACAGACTGTGAGAGTGGAGATTGGATGCAGGCTTATGCAGATTACCTTGTACAGTACTGGAAGTACTACCGCAATGCTGGTGTACCTCCGACCCATCTTGGTTTCATAAATGAGCCTGAAGAAGTCGTATCCTATGCTAGTATGGAAACGAATGGTACTCAGGCAGCTGAATTTATTAAAGTCCTTGGACAGACACTTGAACGGGAAGGAATTGACATAGAATTGACCTGTTGTGATAGCAATGGTTGGCAGCATCAGGAGAATTTAATGGCTGGCTTGCAGGCAGTGGGACCAGACGGCAAGAGTGGCGAAGACTACCTCTCTGTGATTACTGGCCATGGCTACGCGTCGCCGCCTAACTTTCCCCTTAGTACGAAACTTCGTACCTGGCAAACAGAGTGGACCGACTTGTCTGGGCACTATACTCCTTATACCTTCTACAGTACTGGGGCGGCAGGAGAAGGACTAACCTGGGCTGAAAATATTCAGACTGCATTTGTAAATGCCAACGTAAGCGCATTCTTGGGTTGGATTGGCGCGGAGAATGCAACCGTTAACTCAGGATTGATTACGCTTCTCGGGGACGAAATCGTCCCCTCCAAACGATTTTGGGCGATGGCGTCTTTCAGCAAATTTGTTCGGCCAGGTGCAGTACGAGTTGAGAGTACATCCTCTGACCCATCGCTGAAGGTTAGTGCCTTCCAAAATAAGGATGGAATCATTGCCTTACAAGTGATTAACAACAGTACATCCAGCGCGGCAATGTCTATTGATTTCGGAGTACCGGTACGTGGAAATCATCAGTGGAAATATGTTGTTCCTTGGATCACAAATAATGATCATGATCTCGACGCCCAGAAGAGTATTCCTATTGGTAAAGATGGGAAATTTGAGGCAGTAGTGCCTTCGAAGTCATTAGTGAGCTTTGTTTCGAGTCACAAG

**SlXyn10A** (*Scheffersomyces lignosus*)

Gene ID: BCGS01000004.1|-processed-gene-12.20

>genemark-gi|1002316369|dbj|BCGS01000004.1|-processed-gene-12.20-mRNA-1 gene=genemark-gi|1002316369|dbj|BCGS01000004.1|-processed-gene-12.20 CDS=1-1077 - extracellular **ATGAAGTTAGAACTTGCTTCCGCTTTCCTCATCCTCAGTGGCCTTGCTTCTGCC**AACCCTATCCCAAAGGGAGGCAGCAAGAACGCTTGTCAGACTCCAGCCGCTAACCTTAATGAACTTGCTGTTGCTGCTGGTAAGTTGTACTTTGGTACTGCTACTAACCAAGAGCAGTGGAATGACACTAACTATGTCAACATCATGCTTGACCAATTTGGTTCTCTTACTCCTGCTAACGTTCAAAAGTGGCAGTTTACTGAACCTGAACAAGGTGTTTTCAACTACACCCTAGGTGATGAGTTTGCTGATTACGCCTTGAAGAACAAAAAGGTACTCTTATGTGACACCCTTGTCTGGCATCAGGAATACCCACCTTGGTTAGACCAGAAGAAGTGGACCAAGGAGGAGTTGCTTAAGGTTATCGACCAGCACGTTTACTCTGAAGTCTCTCACTTCAAGAACAGATGTTTCTCCTGGAACGTCGTCAACGAAGCTTTGAATGACGATGGTACTTGGAGAGAAACCCTCTTCTACAATGTCACCGGTACTGATTACATTGCTACTGCTTTCAGAGCCGCTGCTCGTGCCGACCCTAAGGCTCAACTTTACTACAATGACTACAACATTGAGTCCCCAAGTGACAAGTCAACCGCAGTTGAGAATATGATCAAGACCCTTAAAAAACAGAAAGTCAAGATTGATGCTGTTGGTCTTGAGTCCCACTTCATTGTAGGTGAGACCCCATCTGAGGCATCACAACAACAACAAATGCAAGCTTACATTGACTTAGGTGTTCAAGTTGTTGTTTCTGAACTTGATGTTAGATTCCTGGCCCTTCCACCAACTGAAGCCGGTCTCGTTCAACAAACTAACGACTACCAATCCTCTGTCAACGCTTGCATCAAGGTTGGTAAGCAATGTATGGGTATCTCTGTCTGGGACTTTGATGACGCTTACTCTTGGATTCCAAGCACCTTTGCTGGCAATGGTGACGGTGACTTGTGGTACGCCAACTTCACCACTGCTCCAGCTTTCGACGGTGTTGTCTCTGCTCTTGAAGCCGGAGAACTCAAGCACAAC

(Bold bases: predicted signal peptide)

***Sl*GH5_22** (*Scheffersomyces lignosus*)

Gene ID: BCGS01000006.1|-processed-gene-0.90

>genemark-gi|1002316367|dbj|BCGS01000006.1|-processed-gene-0.90-mRNA-1 gene=genemark-gi|1002316367|dbj|BCGS01000006.1|-processed-gene-0.90 CDS=1-1452 ATGTCTTCTGGATTTTTAACTACCAAAGGTACTAAGATCGTCGATGGTTCTGGGAAATCCGTCGTTCTTGTCGGAACTTCAATCGCTGGTCATCTTAATATGGAAAATTTCATTACTGGCTACCCAGGACATGAGACTGAGCATAAAAAGGTGCTTAAGAAAAAGATTGGTGCTGAGAAGTTCGACTATTTCTTCGACAAATTCTACGAGTACTTCTGGACTGAGAAGGATGCGGAATTTTACAAGAACGAGTTGGGTTTCAATTGCTTGAGAATTCCATTCAATTACCGTCACTTCATCGACGATGAGGATGACTTGTTCAAGATCCTTCCAAAAGGGTTTGAAAGATTAGACAGAGTTGTTGATATTTGTTCCAAGTATGGTATCTACACTATCTTGGACCTTCACGCTACACCTGGAGGACAAAACCAGGACTGGCATTCCGACTCTGGTATCCACAAGTCACTCTTCTGGGAATTCAAGGTATTCCAGGACTGTATTGCTAATCTCTGGGTCGAGCTTGCCAATCACTACAAAAATAACACTTGGGTTGCAGGTTACAACCCATTGAATGAACCTGCCGTTTCTGACCACAGTAAGTTAGTAAACTTCTACAAGAGATTGGACAAGGAAATTAGAGCTGTTGACCCTAACCATATCTTCTTCCTTGATGCTAACACCTACTCCATGGATTTCAGACAATTCCCAGCCCCGGCTGATTTCGTTAAGAACGCTGTCTATTCCGTTCACGACTATTCCAATTTTGGATTTCCTAACATAGAGGGGACTCTCTACAAAGGCTCCGAGGATGAGAAATCTAAGTTGAAGAGCCAATACGAGAGAAAAATTGAGTACAACAAGAACAATAATGTCCCTGTATGGAATGGTGAATTTGGGCCAGTGTATGCTTCTAAGGAAAGAGGTGACAAAAACCCAGAAATTATTAACAAAGCACGTTACAATGTTTTAAAAGATCAAATGGCCATCTACGCCAAGGGAGATCCTTCTGGAGATGGCTCTCCCATCTCTTGGTCTATCTGGTTATACAAGGACATTGGATACCAGGGTTTGACTTATGTTTCTCCGGATTCGAAATGGTACAAAATATTCGGTTCTTGGTTGTTGAAGAAGAAAAAATTAGGATTAGACAGATGGGGTAACGACATTGATGCAGAATACAACAAGTTGTACGAAAGTTTGGTTAACCACATCGAAGAGAATACTCCGAAAAAGTACAATAGGGTTTTGTACCCTCACGTATTTAGTGTTCAAGATTACTTGTTCAGGGTTACTAAGGACATGTTATTCTCTCAGATAGCCCAACACGAGTATGCTGATTTGTTTGTTGGTTTAAGTTTTGAGGAGTTAGATGAGTTGGCAGCTTCGTTCAAATTCGAGAACATAGTGCAAAGAAAGGAATTGAACCAAATTTTGAAAGAGTATTCCCAAATT

***Wc*Xyn5_22A** (*Wickerhamomyces canadensis*)

Gene ID: ID_6015-processed-gene-0.66

>genemark-NODE_47_length_66922_cov_25.6309_ID_6015-processed-gene-0.66-mRNA-1 gene=genemark-NODE_47_length_66922_cov_25.6309_ID_6015-processed-gene-0.66 CDS=1-1434 ATGTCTGGTTTCTTGAAAGTGAAAGGTACTAAAATTGTGGATGAAAACGATACTCCAGTTGTTTTGAAAGGCACTGCCAATGGTGGCCACTTAAACATGGAAAATTTCATCACTGGTTATCCAGGTCATGAGACTGAACACAAGAAAGTCTTGAAGTCGAAGATTGGTGAGGAGAAGTTCAACTTCTTTTTTGACAAATTCTATGAATATTTCTGGACAGAAAAAGATGCTATTTTCTACAAGGAGGAACTGAACTTCAACACCTTGAGAATTGCCTTCAACTACAGACACTTTTTAGATGATGACGGTGATCTGTTTGAGATCAATCCAGTTGGTTTCAAAAGGTTAGATGCTATTGTCAATACTTGTGCAAAACATGGCATTTACACAATCTTGGATCTTCATGCTGTTCCAGGTGGTCAAAATCAAGATTGGCACTCAGACTCATCGATCCATAAAGCTTTGTTCTGGGAGTTCAAGGTATTTCAAGATGCCATTGTCAATCTTTGGGTGAAAATAGCGGAATATTACAAAGACAATACCTGGGTTGCGGGTTATAATCCGTTAAATGAACCTGCAGTTTCCGATCATTCAAAGCTCGTCAAATTTTATGAAAGAGCTGAGAAGGAGATTAGAAAAGTGGATCCAAATCACATTTTATTCCTGGATGGTAATACATATGCTATGGACTTCCGTCAATTCCCTGATGAACCATTACCAAACACCGTATACAGTATTCATGATTATTCAGTGTTTGGATTCCCAAATCCTGAAGGTAAGTTATACCAAGGTACGGCAGATGAAAAAGCAAAAATCAAAAGCCAATATGAACGTAAGATTGAATATCAAAAGAAGAATAAGGTTCCCGTTTGGAATGGTGAATTCGGTCCAGTTTATGCCTCTACAGAACGTGGTGATTTTAACATTCAAGCGACTAATGAATCCCGCTACAAGGTTTTGAAGGATCAACTGGAGGTTTATAAACACGGGGATCCTTCTGGTGATGGATCACCAATCAGTTGGTCTATTTGGGTTTACAAAGATATTGGGCTCCAAGGTTTAACATATGTTAATCCAAATTCCAAATTCTTTGAGATATTTGGTTCTTTCCTGTTGAAGAAAAGAAAACTTGGATTGGATAGATGGGGTAATGATATTGATCCAGCTTATGCCAAGTTATATGATGATCTCCAAGCTCATTTTCAGGAAAATATCCCAGAAAAACATCATCATGCTTTATATCCACATCTTTGGTCAATCAAGGATTATATCGCTCGTTCAGTTAGGGATACTTTATTCTCACAATATGCTCAACATGAATATGCTGACTTGTTTGTTGGTTTGAGTTTTGAGGAACTAGATGAGCTAGCTGCTAGTTTCAAGATTGAGAACGTTTCCAAGAGAGAAGAGTTGAACGCTATCTTGAGAGCTGCT

***Wc*Xyn5_49A** (*Wickerhamomyces canadensis*)

Gene ID: ID_1891-processed-gene-1.61

>genemark-NODE_6_length_405598_cov_26.5625_ID_1891-processed-gene-1.61-mRNA-1 gene=genemark-NODE_6_length_405598_cov_26.5625_ID_1891-processed-gene-1.61 CDS=1-1512 ATGGGATTATTAGATAAGTTCAAGGATCTTGCATTGGACCACGAAGAGTCTGCACTAAAGCCTGCTGGTCCTGCTGCTCCGGTTGGCAAAGCTCCAGACAAGAAGTCTATATATCAAAACAGATTCAACTTTGGTGTCAATTTTGGTTCTCTATTCGTCCAAGAGAAGTTCATATTCGATAGGTTTTTTCCTGATGATACACATACTGAACTCGACGGTGTCACTGCTTACATAAAGGAGAATGGAACAAAGAAAACTATGGAAGATTTGGAGAATCATTGGAATGGATATGTCACCGATCAAGACTGGGAGTGGCTCAGAAGTAAGGGTGTCACTGCTATCAGAATCCCCATTGGTTATTGGCATGTCAATGGGGGTTTCTACACCAAGGGAACCCCATTTTTGGCCATCGCATCTGTTTATAAGAACTCCTGGGTGATCTTCAAGAACATTGTTGCAAAGGCAAAGCAGTACGATATAGGTGTCCTTGTGGATCTACATGCCCTCCCAGGTGGTGCTAATACCGCTGAACACAGTGGTGTTCAATTGAAACAAGCGTCATTTTGGACTGAGAAGAAGTCAGAAACATTGGCCCTCTCAATTTTGGAATATGTTGCAGATGAGCTAAAAAACGAGGAGAACGTTATTGGACTACAAATTGTCAATGAGTCAGAATTTGACAATAATGCAAGTGCACAAAAACGCTATTATGCAAATGCTGTTAAGAAAATCCGCAAAGTGGACCCTAATCTACCTATAATTATCTCAGATGGATGGTGGACCGATCAATGGGTCAAATGGATCAACGAGAATGAAGCCAATTTCAAAGGTCATGGGTCGTCTGGATTAGTCATCGATACACATGTCTATCGTTGTTTCTCTGACAGTGATAAAAAGAAGTCCCCTGAGCAGATAATTCGAGACTTGAACGGTGATCTTTTAACTAATTTATCCGGTCCGGCTGATATCATTGTGGGCGAGTATTCACATGTTCTTGATGGTGAGACTTGGAAGAAAAAATCACATGATCAAGGTCAACTTGTGAAACAATTTGGGAATGAACTGGGGAAGCTCTTCAAGCAGCGTGCTAATACGGGATCATTTTTTTGGACATTCAAGTTTCAACATGGAGATGGGGGAGAATGGGGGTTTGTTCCTCAGGTAGAGTCAGGAGCTGTGCCAATCCCTATTTTAGTTGCCAAAAATTCCCCATCAGAGGATGACTTCAGCAAAGCGTTAAATGATGAATTGAATTCACATTCCTCATATTGGAATGACCAAAATAGCAACGAAAATTATGAACATTGGAGATTCAAAGAAGGATTCACCACTGGCTGGGCCGATGCTGTTGCATTTGCAAAATTCAATGGATCAACTATAGGTCGTTACAATGCATGGGCTTCTTCAAGACGAGCTGAACATATCAATCATAGAAACTCAAGTAACTTCCTGTGGGAGTGGGACCAAGGGTTTGGTAAAGGTATTGAAAGGGCACAGCAAGCTATATTTGGT

**Table S1.** Data collection and refinement statistics for the structure of *Bm*Xyn11A (PDB: 8B8E)

| Resolution range | 63.11 - 1.55 (1.605 - 1.55) |
| --- | --- |
| Space group | P 1 21 1 |
| Unit cell | 83.039 37.8599 142.609 90 104.27 90 |
| Total reflections | 240242 (21850) |
| Unique reflections | 125328 (12109) |
| Multiplicity | 1.9 (1.8) |
| Completeness (%) | 98.92 (95.31) |
| Mean I/sigma(I) | 4.42 (0.85) |
| Wilson B-factor | 18.25 |
| R-merge | 0.07084 (0.7017) |
| R-meas | 0.1002 (0.9923) |
| R-pim | 0.07084 (0.7017) |
| CC1/2 | 0.993 (0.425) |
| CC* | 0.998 (0.772) |
| Reflections used in refinement | 124608 (11892) |
| Reflections used for R-free | 1995 (197) |
| R-work | 0.1929 (0.3491) |
| R-free | 0.2255 (0.3434) |
| CC(work) | 0.955 (0.688) |
| CC(free) | 0.940 (0.567) |
| RMS(bonds) | 0.013 |
| RMS(angles) | 1.08 |
| Ramachandran favored (%) | 97.57 |
| Ramachandran allowed (%) | 2.43 |
| Ramachandran outliers (%) | 0.00 |
| Rotamer outliers (%) | 0.51 |
| Clashscore | 1.95 |
| Average B-factor | 22.02 |

Statistics for the highest resolution shell are shown in parentheses.

**Table S2.** Gradient program for separation of xylooligosaccharides using ion chromatography

| **Gradient** | **Time (min)** | **300 mM NaOH (%)** | **1 M sodium acetate (%)** |
| --- | --- | --- | --- |
| Equilibration | -5 to 0 | 15 | 0 |
| Injection | 0 |  |  |
| Gradient 1 | 0 to 10 | 15-33 | 0 |
| Gradient 2 | 10 to 20 | 33 | 0-12.5 |
| Gradient 3 | 20 to 22.5 | 33 | 12.5-66 |
| Re-equilibration | 22.5-25 | 33-15 | 66-0 |


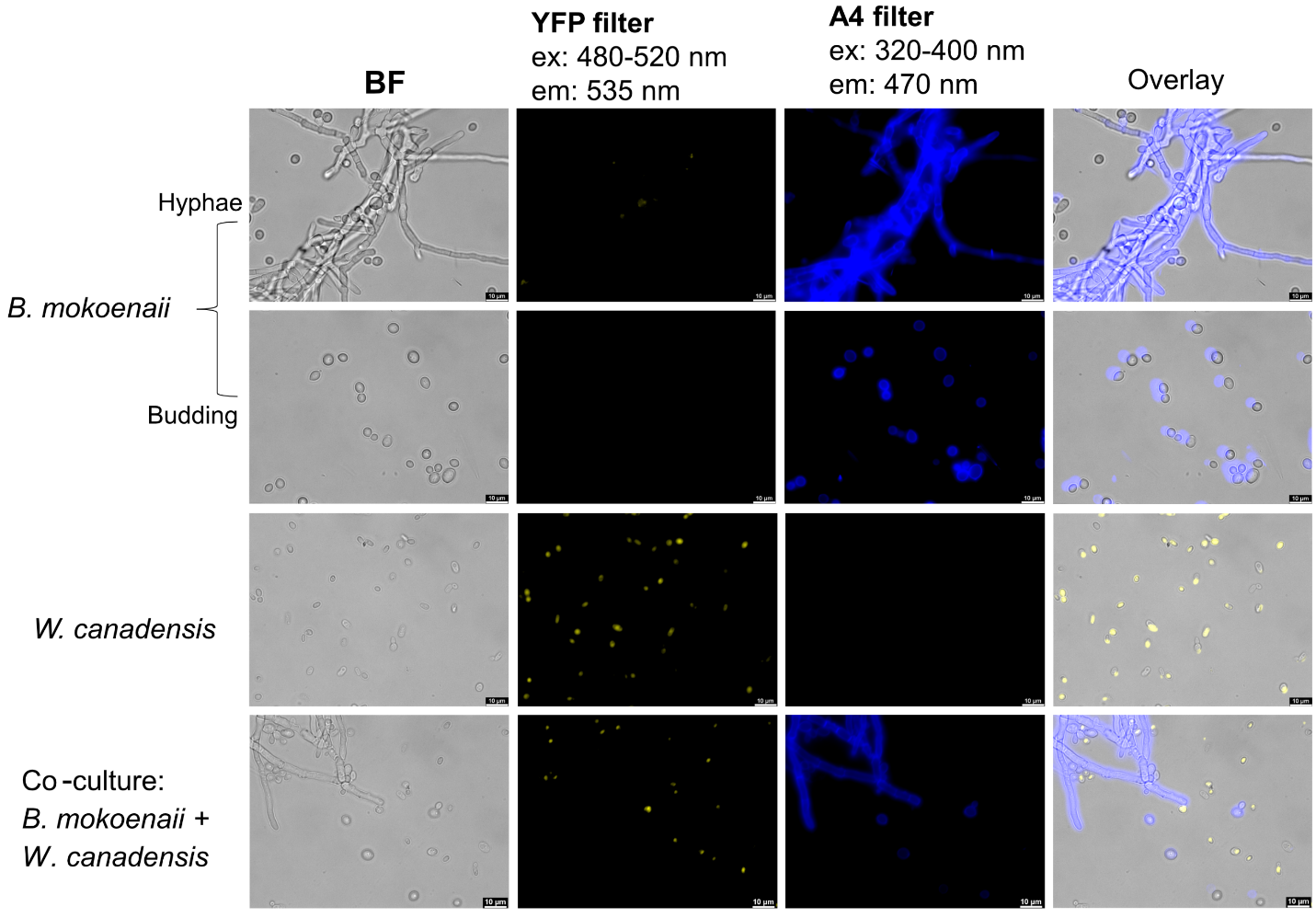


**Figure S1.** Fluorescence staining of *Blastobotrys mokoenaii* and *Wickerhamomyces canadensis* using FUN-1 and Calcoflour white. Yeasts were grown in YPD and stained either separately or together with FUN-1 and Calcoflour white. Scale bar = 10 µm.


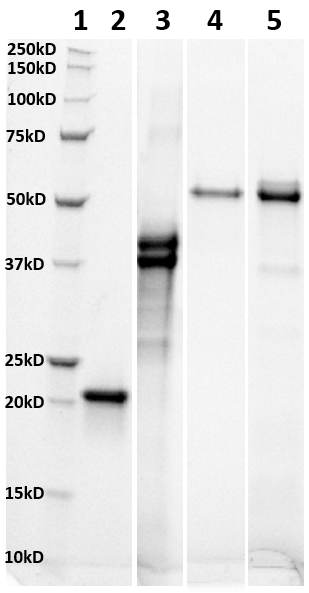


**Figure S2. SDS-PAGE of purified proteins. Lane** **1:** Molecular weight. **Lane 2:** *Bm*Xyn11A (22.37 kDa). **Lane 3:** *Sl*Xyn10A treated with endo H (= 39 kDa) **Lane 4:** *Wc*Xyn5_22A (55.56 kDa). **Lane 5:** *Wc*Xyn5_49A (57.01 kDa). Expected protein molecular weights: *Bm*Xyn11A= 22.37 kDa, *Sl*Xyn10A = 39 kDa, *Wc*Xyn5_22A = 55.56, *Wc*Xyn5_49A = 57.01 kDa. In-gel proteomics of *Wc*Xyn5_49A confirmed bands at 57 kDa and 37 kDa were both the recombinant protein.


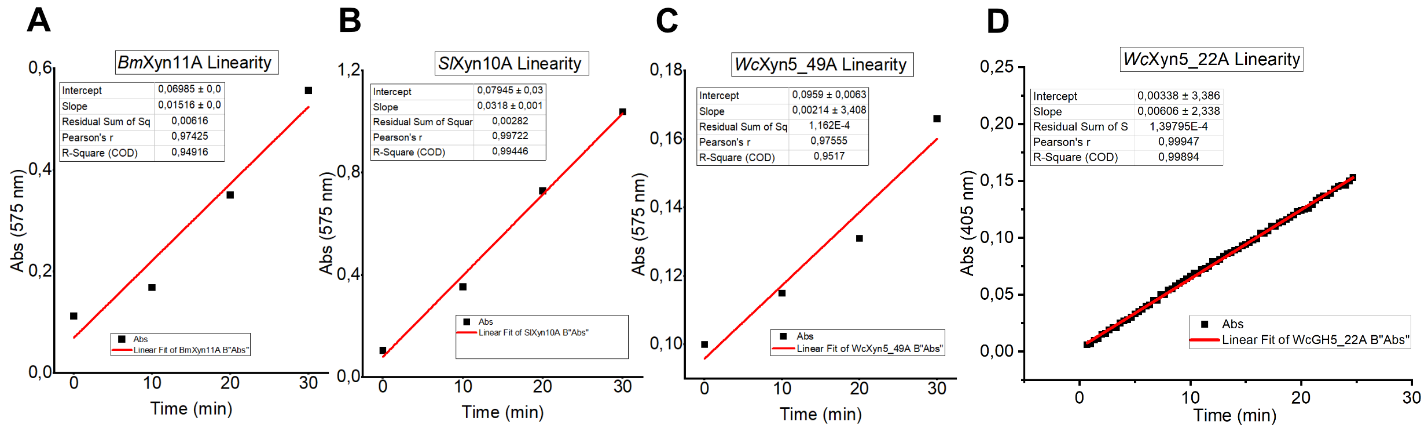


**Figure S3. Linearity of recombinant enzyme** **activity in xylan.** (A-C) Activity was determined by DNS assay for *Bm*Xyn11A, *Sl*Xyn10A, *Wc*Xyn5_49A. (D) Activity for *Wc*Xyn5_22A was determined with *p*NP-β-d-xylose. Recombinant enzymes were used directly from the final purification except *Bm*Xyn11A which was diluted 1000x.


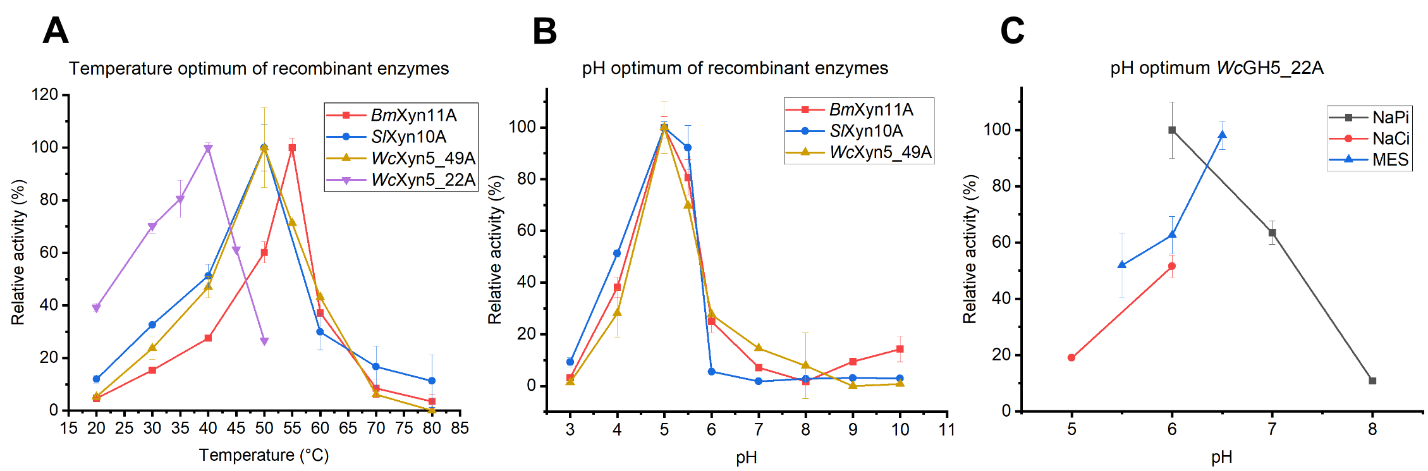


**Figure S4. Temperature and pH optima for recombinant yeast enzymes.** (A) temperature optima of recombinant enzymes. (B) pH optima of recombinant enzymes. (C) pH optimum using 2.5 mM *p*NP-β-d-xylose was used for *Wc*Xyn5_22A in different buffered systems. For *Bm*Xyn11A, *Sl*Xyn10A and *Wc*Xyn5_49A, temperature and pH optima were determined using 1 % beechwood glucuronoxylan and DNS assays.


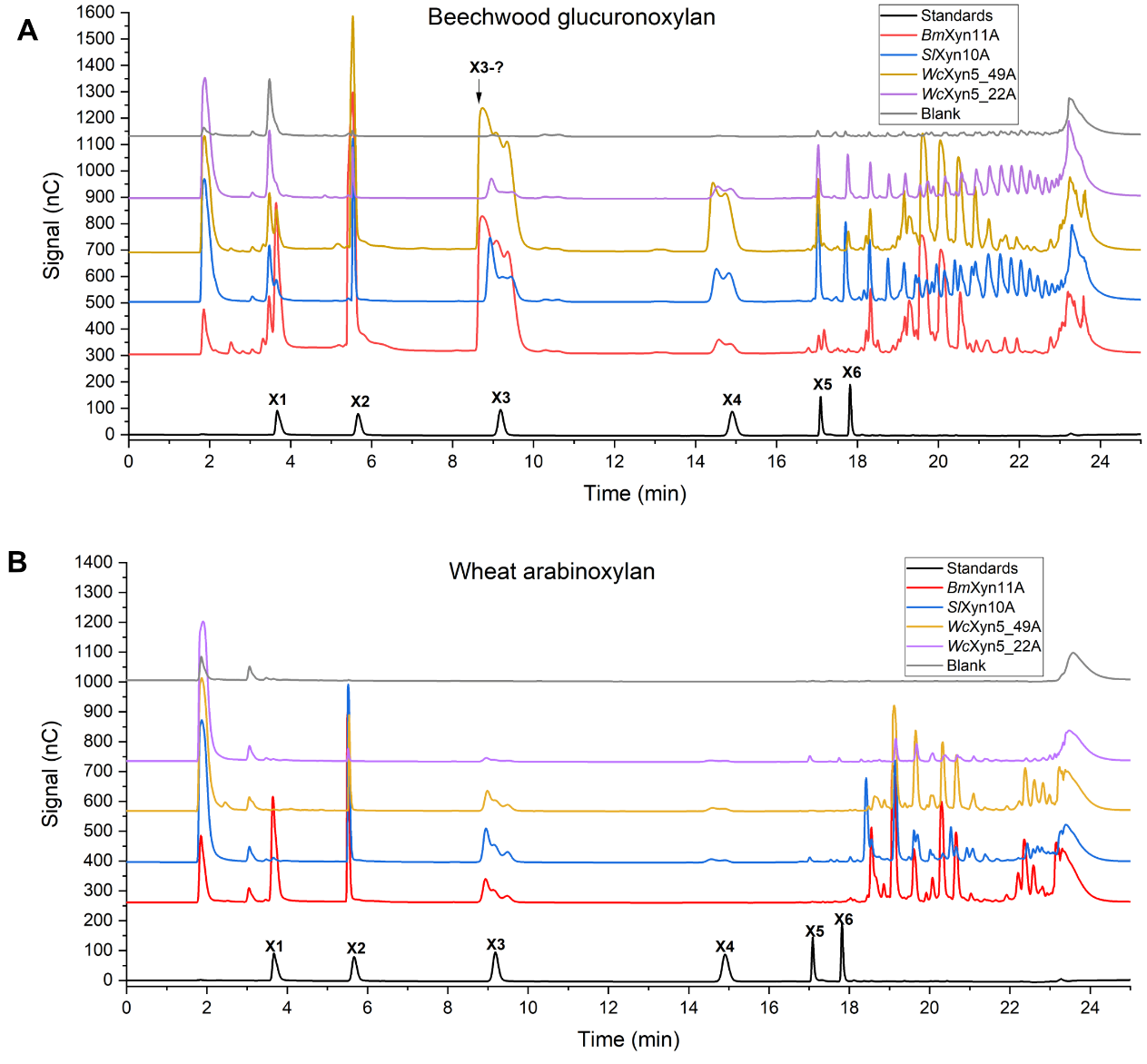


**Figure S5. HPAEC-PAD chromatograms enzymatic hydrolysis of xylans after 24 h.** (A) Chromatogram of beechwood glucuronoxylan hydrolysis profiles. (B) Chromatogram of wheat arabinoxylan hydrolysis profiles.


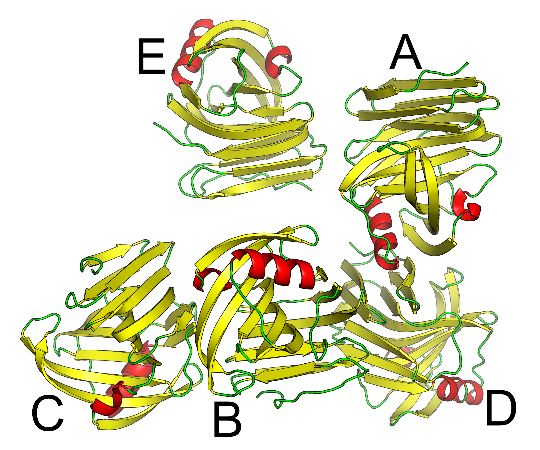


**Figure. S6.** Overall structure of the asymmetric unit of newly solved *Bm*Xyn11A (PDB: 8B8E). The chain IDs are labelled A-E. The secondary structure features are colored – β-sheets, yellow; α-helices, red; loops, green.


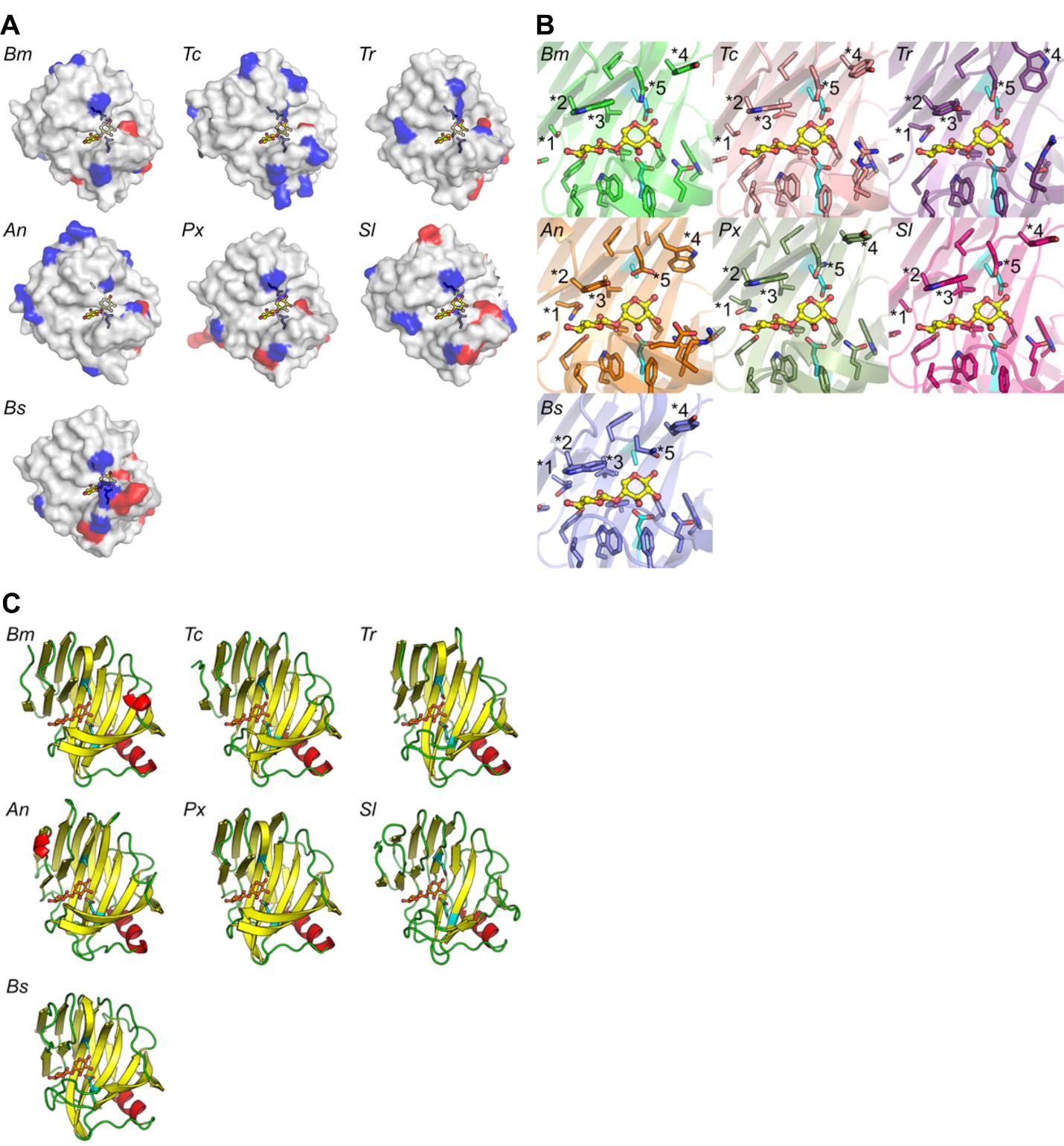


**Figure S7.** Comparison and diversity of GH11 family enzymes from various kingdoms. Shown are enzymes from *Blastobotrys mokoenaii* (*Bm*, yeast)*, Talaromyces cellulolyticus* (*Tc*, fungi)*, Trichoderma reesei* (*Tr*, fungi)*, Aspergillus niger* (*An*, fungi)*, Paenibacillus xylanivorans* (*Px*, bacteria), *Streptomyces lividans* (*Sl,* bacteria)*, Bacillus subtilis* (*Bs*, bacteria). (A) Surface representation showing distribution of charge on the GH11 enzymes. (B) Comparison of active site tunnels. Amino acid positions shown with an asterix (*X) indicate observed differences between GH11 members which may indicate evolutionary clades in the enzyme family. GH11 xylanases possess a Ser residue in place of the more common Asn, which is intriguing as this position may be involved in directing the substrate into the active site (indicated with *1). The GH11 enzyme from *T. cellulolyticus* possesses a Thr instead of Val which might modify the entry and binding of substrates (*2). Some fungal enzymes have swapped the positions of Tyr and Trp residues (*3 and *4), and all of the fungal enzymes have Asp instead of Asn (*5). (C) Secondary structure comparison between the same enzymes. The superimposed substrate is shown as an orange ball and stick representation. In all figures, the superimposed substrate is overlaid from the *Aspergillus niger* structure (PDB: 2QZ2, crystallized with xylopentaose).


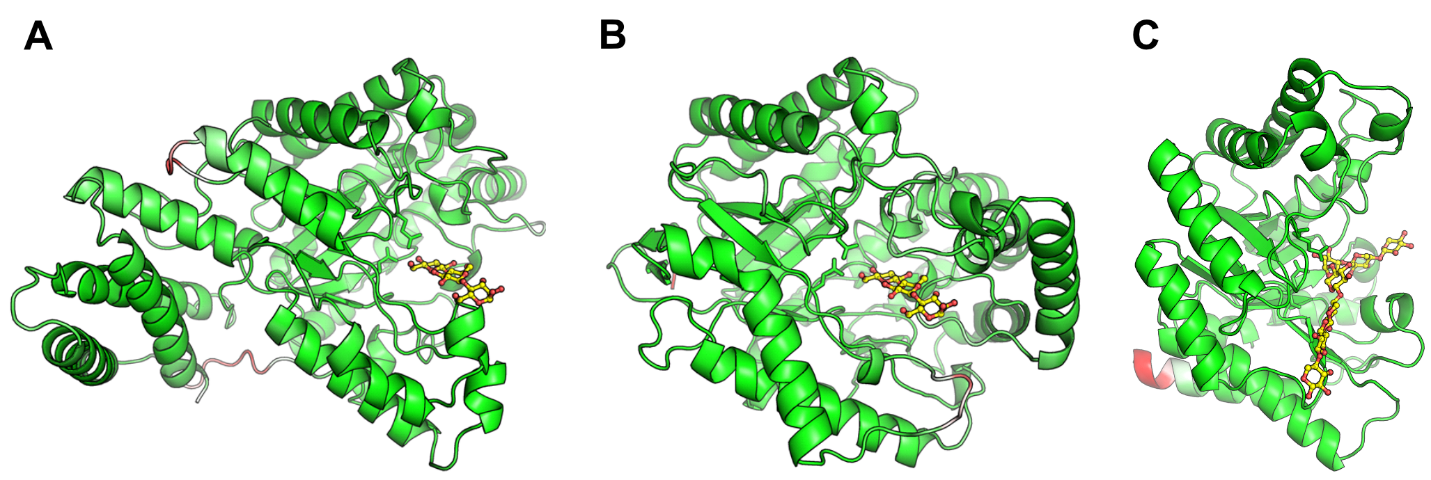
**Figure S8.** pLDDT prediction scores of AlphaFold structures (Fig. 6). The color scale shows the value of the pLDDT at each Cα from red (70) to green (100). No regions had a score below 70 (which would indicate low model confidence). (A) *Wc*Xyn5_49A, (B) *Wc*Xyn5_22A, (C) *Sl*Xyn10A.
